# Management of patellofemoral joint osteoarthritis using biomechanical device therapy: a systematic review with meta-analysis

**DOI:** 10.1186/s13643-021-01708-3

**Published:** 2021-06-09

**Authors:** Michael J. Callaghan, Elizabeth Palmer, Terence O’Neill

**Affiliations:** 1 Faculty of Health and Education, Department of Health Professions, Manchester Metropolitan University, Manchester, UK; 2 Manchester University NHS Foundation Trust, Manchester, UK; 3 Centre for Epidemiology Versus Arthritis, Faculty of Biology, Medicine and Health, Manchester Academic Health Science Centre, The University of Manchester, Manchester, UK; 4 Therapies Department, Aintree University Hospital, Liverpool, UK; 5 NIHR Manchester Biomedical Research Centre, Manchester Academic Health Sciences Centre, Manchester University NHS Foundation Trust, Manchester, UK; 6 Department of Rheumatology, Salford Royal NHS Foundation Trust, Manchester, UK

## Abstract

**Background:**

Current clinical guidelines recommend conservative management including non-pharmacologic therapy prior to considering surgery for knee OA. There is a paucity of clinical trials investigating the use of biomechanical device therapies on those with patellofemoral joint osteoarthritis (PFJOA). The aim was to systematically review the effectiveness of biomechanical devices (bracing, taping, and footwear) in the management of symptomatic PFJOA.

**Method:**

The Cochrane, PEDro, MEDLINE, CINAHL, AMED and EMBASE electronic databases were search from inception to October 31, 2020. Included studies were randomised controlled or clinical trials studying any form of biomechanical device therapy in the management of PFJOA in the English language. Studies included in the search were quality-appraised using the PEDro scoring system.

**Result:**

Eleven studies were identified which included assessment of either patellar taping, or foot orthotics, knee bracing or combined physiotherapy treatments. Trial quality ranged from ‘poor’ through ‘fair’ to ‘good’. For patellar bracing, pooled analysis of two good quality randomised controlled trials showed no overall significant improvement on a visual analogue scale (VAS) (random effects (RE) standardised mean difference (SMD) = −0.42 (95%CI −1.12 to +0.29).

Pooled data from the same two studies showed a non-significant improvement in favour of bracing assessed by the KOOS/WOMAC (RE SMD = −0.18 (95%CI −0.66 to +0.31). Two studies of ‘fair’ and ‘good’ quality applying patellar tape showed a significant reduction in pain immediately after application and after 4 days. A randomised trial of a foot orthotic showed a non-significant improvement in pain after 6 weeks with a between groups adjusted mean difference for maximum VAS of 21.9 mm (95% CI − 2.1 to 46.0) and 8.1 (95% CI− 6.9 to 23.1) for KOOS pain. A multimodal physiotherapy intervention (which included taping in two studies) showed a pooled significant improvement in VAS (SMD = −0.4; (95% CI −0.71 to −0.09) at 3 months compared to controls.

**Conclusion:**

There is some good quality evidence that a combined physiotherapy approach significantly reduces short-term pain in those with PFJOA. Long-term effects of all interventions are still unknown, which indicates the need for further research to determine the longer term impact of all biomechanical devices on outcomes in symptomatic PFJOA.

**Supplementary Information:**

The online version contains supplementary material available at 10.1186/s13643-021-01708-3.

## Background

The patellofemoral joint (PFJ) is an important source of symptoms in knee osteoarthritis (OA) compared to the tibiofemoral joint (TFJ). In symptomatic knee OA cohorts, the prevalence of radiographic PFJOA is 57% and in radiographic and symptomatic knee OA cohorts the prevalence of PFJOA is 43% [1]. Typical features of PFJOA are anterior knee pain or retro-patella pain aggravated with PFJ loading activities such as stair ambulation, squatting, kneeling or rising from sitting. PFJOA symptom management by conservative treatment is critical [2]. Altered mechanics and increased PFJ stress are thought to be involved in the progression of PFJOA [3]. Therefore, patellar bracing and patellar taping (in which medical or sports tape is applied either onto or around the patella) have been suggested as interventions which alter patella position in the trochlea, reduce PFJ stress and limit structural damage [4]. Bracing has the advantage of being easier to self-apply than taping without loosening [5].

Current guidelines in the UK, Europe and America recommend surgery for knee OA only after conservative management including pharmacologic and non-pharmacologic therapy has been exhausted [6–9]. Biomechanical device therapy, which includes taping, bracing and modified footwear with orthotics, insoles or inserts, is appealing as it is not linked with any of the systemic adverse effects associated with pharmacological therapy. There are a paucity of clinical trials to support or refute the use of various biomechanical device therapies for the management of PFJOA with a greater number of clinical trials and systematic reviews focused on TFJ OA [10]. Such data are important to inform optimum clinical practice for this large, symptomatic patient sub-group. An international consensus statement on PFJOA produced a narrative review which implied further need to evaluate treatment outcomes in people with PFJOA [11]. Our aim was to undertake a systematic review with meta-analysis of the efficacy of biomechanical device therapies in the management of PFJOA.

## Methods

### Search strategy

The MEDLINE, AMED, EMBASE, CINAHL and Cochrane Database of Systematic Reviews electronic bibliographic databases were searched from inception to October 31, 2020. The search strategy and search terms are presented in the supplementary file. Search terms were adapted to the requirements of each specific database. Where the full text was obtained, reference lists were searched. Inclusion criteria were English language, peer reviewed, original research, randomised controlled or clinical trials studying biomechanical device therapy (brace, taping and modified footwear including shoe inserts, insoles or foot orthotics) in the management of PFJOA, with validated outcome measures. Exclusion criteria included studies where subjects did not have predominant PFJOA, systematic and narrative reviews, clinical commentaries, editorials or studies from non-peer reviewed journals. Reference lists of the full-text articles were also checked to ensure any articles not captured in the electronic search were included. We searched the grey literature guided by the ‘Grey Matters’ checklist (Grey Matters: a practical tool for searching health-related grey literature (https://www.cadth.ca/resources/finding-evidence). The grey literature search included a search of clinical trial registries (Cochrane Central Register of Controlled Trials with ClinicalTrials.gov) Google Scholar, and forward searches of SCOPUS/Web of Science for all included articles as well as searching of the reference lists of included articles and related systematic reviews.

### Assessment of study quality

One author (EP) initially independently checked the titles and abstracts of the articles against the inclusion and exclusion criteria. Non-eligible studies were excluded. The methodological quality of full texts was independently assessed by two authors (EP and MJC) using the Physiotherapy Evidence Database critical appraisal tool (PEDro http://www.pedro.org.au). Trials were awarded one point if each criterion was clearly satisfied. Criterion 1 was not included in the final PEDro score, so each study had a possible maximum score of 10. We considered total PEDro scores of 0–3 as ‘poor’, 4–5 as ‘fair’, 6–8 as ‘good’, and 9–10 as ‘excellent’ [12]. Any discrepancies in the appraisal scores were resolved by discussion and consensus, after which an agreed score was allocated (Table 1).

**Table 1:** Summary of the PEDro appraisal criteria for all none studies

PEDRO criteria												
Studies	1	2	3	4	5	6	7	8	9	10	11	PEDro Score
[13]	X	X	X	X			X	X	X	X	X	8
[14]	X	X	X	X			X	X		X	X	7
[15]	X	X	X	X				X		X	X	6
[16]	X	X		X			X		X	X	X	6
[17]	X	X		X	X		X	X	X	X	X	8
[18]	X	X					X	X		X	X	6
[19]	X	X	X	X	X		X		X	X	X	8
[20]	X	X						X				2
[21]	X	X	X	X			X	X	X	X	X	8
[22]	X	X	X	X			X	X	X	X	X	**8**
[23]	X	X	X	X			X	X	X	X	X	**8**

**Key:** **X:** point awarded when a PEDro criterion was clearly satisfied. Note – item 1 (eligibility criterion) is not included in the overall PEDro score

The number corresponds to the following PEDro criteria: 1=Eligibility criteria stated, 2=Random allocation, 3=Concealed allocation, 4=Baseline comparability, 5=Blinded subjects, 6=Blinded therapists, 7=Blinded assessors, 8= at least 85% follow-up, 9=Intention-to-treat analysis, 10=Between group statistical analysis, 11=Point measures and measures of variability

### Data extraction

The study design, sample size, exclusion and inclusion criteria, participant characteristics including age, BMI and sex were extracted from the studies. Data extracted to measure the effect of the devices were from visual analog scales (VAS) and numerical rating scales (NRS), specific outcome tools (KOOS, WOMAC), the perceived global rating of change (GRoC), and biomechanical data from knee joint position and muscle strength. Additionally, details on the type and frequency of intervention, comparators and outcomes were collected and recorded by one author (EP) and checked independently by a second (MJC).

### Statistical analysis

For each analysis, statistical heterogeneity was evaluated using the inconsistency value (*I*^2^). An I^2^ of 75% and above was interpreted as high heterogeneity [24]. A random-effects meta-analysis was adopted for all continuous data outcomes. All analyses were conducted using RevMan v.5.3.5 software (The Nordic Cochrane Centre, The Cochrane Collaboration, (2020) www.revman.cochrane.org).

## Results

### Search results

**Fig. 1:**
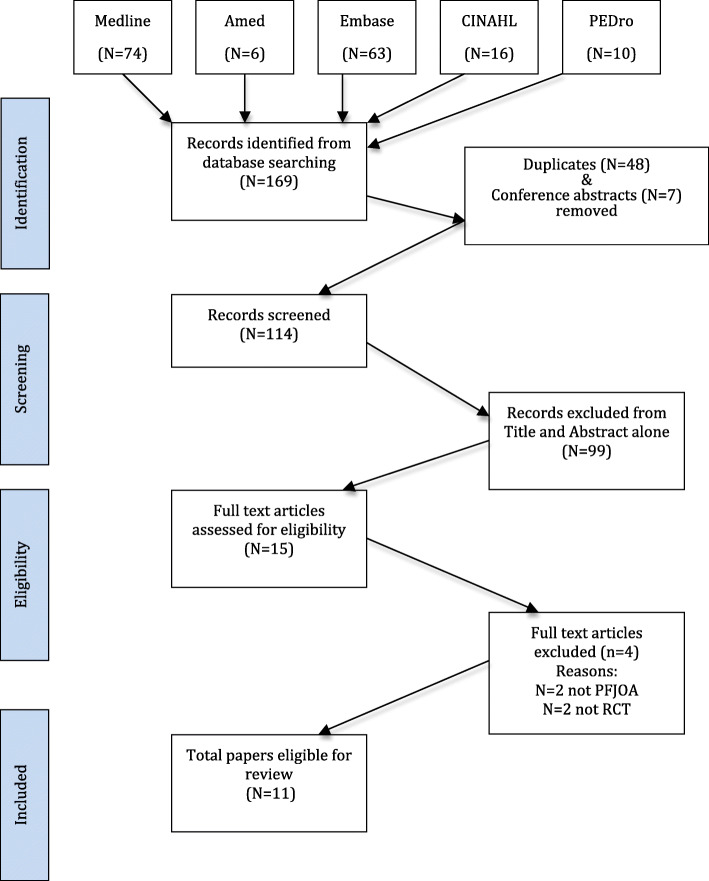
PRISMA diagram

The combined search from all of the databases produced 167 articles and following removal of duplicates and conference abstracts, 115 articles remained. 99 articles were discarded based on the title or the abstract alone. From the remaining 16 full texts, another five articles were excluded. Two were excluded as the participants were not randomly allocated to a brace condition [5, 25], two others because the participants either did not have PFJOA [26], or had TFJOA rather than PFJOA [27, 28]. There were no additional articles found from the reference list of the full-text articles (see Fig. 1).

### Methodological quality of the included studies

A total of eleven randomised clinical trials were assessed for methodological quality. The PEDro scores are detailed in Table 1. Nine studies were classed as ‘good’ quality, one was classed as ‘fair’ quality, and one was classed as ‘poor’ quality.

### Study characteristics

The eleven randomised clinical trials had a total of 658 knees with predominant or isolated symptomatic PFJOA. Two studies ([14] (n=30) [15]; (n=106)) assessed some of the participants from a parent trial ([13], n=126). Tan et al. [23] was a biomechanical study nested within a pilot RCT [22]. Two studies did not provide information on the sex of the participants (Kumar & Ganesh 2011 [20];), so excluding these, the overall percentage of females (62%) was greater than males though there was wide variation in the proportion of males and females between the studies (males 14–45% and females 55–86%). The mean age of participants was 53 years (range 55–70 years), and the mean BMI was 28.9kg/m^2^ (range 27.6–31kg/m^2^), with one study not providing participants’ ages [20]. Radiographic knee OA was assessed and described in eight studies all of which used axial (skyline) plain radiographs as a means of identifying participants with either isolated PFJOA or OA that was more severe in the PFJ than the TFJ. Three studies [13–15] included subjects with Kellgren and Lawrence (K-L) scores of grade 2 or 3 in the PFJ, which was greater than the K-L grade for the TFJ, and excluded those whose K-L grade was greater in the TFJ. Those with K-L scores equal in all compartments were included if the signs and symptoms were predominantly in the PFJOA. Two studies [16, 17] used the disease severity based on the Osteoarthritis Research Society International (OARSI) Atlas for Radioanatomic Positioning of the Knee. Both studies included radiographic evidence of either an osteophyte of severity grade equal to or greater than 2, or joint space narrowing (JSN) of 1 with concurrent grade 1 osteophyte(s), in the PFJ on skyline radiograph. They excluded individuals with medial more than lateral PFJ osteophytes or with a K-L grade greater than 2 in the TFJ. Quilty et al. [21] included those with K-L grading grade 3 or with greater osteophytes in the PFJ and the absence of K-L grade 3 or 4 TFJ involvement. [19] used the OARSI atlas to assess JSN and osteophytes. PFJOA was defined as an osteophyte or JSN of grade 2 or greater. Those with concomitant TFJOA were not excluded if symptoms were predominantly PFJOA. Cushnaghan et al. [18] had participants with radiographic evidence of PFJOA (defined as definite joint space narrowing with osteophytosis) predominating in the lateral PFJ facet in all 12 patients with radiographic TFJOA also present in those patients. Kumar & Ganesh [20] stated that their participants were radiologically diagnosed as patellofemoral arthritis, but provide no details of the radiographic technique used. Two studies [22, 23] used NICE clinical guidelines which stipulated that imaging was not required for a clinical diagnosis of OA. One study did not report level of symptoms [21].

All studies identified their recruitment sources. Four recruited from primary care practitioners, hospital physiotherapy departments or from patients’ self-referral through adverts [13–15, 20]. Quilty et al. [21] recruited from a large established community cohort study. Crossley et al. [17] recruited solely from primary care physiotherapy practices. Cushnaghan et al. [18] recruited from a hospital rheumatology clinic. Hunter et al. [19] and Crossley et al. [16] both stated they recruited from the community but did not provide further details. Tan et al. [22, 23] recruited via print media in a variety of locations such as pharmacies, community notice boards, doctors’ surgeries, and allied health professionals’ clinics.

### Randomisation and concealment

Sealed opaque envelopes were used to randomise subjects in three studies [13–15]. Five trials used computer-generated randomisation [17, 19–23]. Two studies did not provide information that their groups were balanced at baseline with respect to the main confounding variables [18, 20]. Five studies used concealed allocation; the other studies did not (Table 1).

### Intention-to-treat analysis

Intention-to-treat analysis was performed in five studies. The crossover design studies [14, 15] had no dropouts as well as those of within subjects design [16, 18, 23]. As all participants completed these studies, a point was allocated in the PEDro score in this criterion. Communication with the lead author in the Crossley et al. [16] study clarified that all 14 participants completed their study [22]. had no dropouts after 6 weeks, but excluded 2 participants in the case-complete analysis.

### Blinding

Due to the inherent nature of all the included studies, it was not possible to blind therapists to treatment allocation. Additionally, only three studies had participants blinded to the treatment groups [17, 19, 22]. In the eight other studies, blinding of the participants was not possible and it is likely they were able to distinguish between the interventions. The assessors were blinded to group allocation in seven out of the eleven studies which would mitigate against, though not exclude the possibility of bias.

### Interventions

The interventions used in the trials were bracing (n =4), taping (n= 2), foot orthoses (n=2), and a multimodal treatment package (which included exercises and taping) delivered by a physiotherapist (n =3). Details of the included studies are outlined in Table 2.

**Table 2:** Summary of randomised clinical trials included in this review

Author	Type of study	Sample size	Participants	Intervention	Comparator	Outcome measure	Results
**Callaghan et al. [**13**]**	Randomised controlled trial	n = 126 M=54 (43%) F=72 (57%)	PFJ OA. Mean age 55.5 years (SD 7.5). K-L score 2/3. PFJ symptoms (stairs/ rising from chair). 3 months of daily pain scoring >40/100 on VAS. Painful palpation of patella facets. BMI=31	Brace with or without strap (participants preference) worn for mean 7.4 hours a day for 6 weeks.	No brace.	VAS (knee pain in last 7 days during nominated activity). Change in PF BML. Secondary: KOOS-pain and KOOS-ADL.	At 6 weeks, between groups adjusted mean differences (VAS: −1.3 cm (95%CI −2.0 to −0.7; p<0.001) PF BML volume: −490.6 mm^3^, 95%CI −929.5 to −51.7; p = 0.03) KOOS-pain 5.7 (95% CI: 0.6 to10.8, p = 0.03 KOOS-ADL 4.5, (95%CI; 0.5 to 8.5, p = 0.03)
**Callaghan et al. [**14**]**	Within subjects crossover design with randomised order.	n = 30 M=13 (43%) F=17 (57%)	PFJ OA Mean age 57 years (SD 7.8). Mean BMI 27.8 (SD 4.2) K-L score 2/3 PFJ symptoms (stairs/ rising from chair) 3 months of daily pain scoring 40/100 on VAS. Painful palpation of patella facets.	Weight-bearing MRI with brace.	Weight-bearing MRI with no brace.	Patella position measured on MRI.	Mean difference between groups Lateral PFJ contact area: (0.94 cm^2^, 95%CI 0.07 to 1.8 p=0.04) PFJ lateral distance −0.06 cm 95%CI-0.12, to -0.01 (p=0.03).
**Callaghan et al. [**15**]**	Randomised controlled trial	n = 108 M=49 (45%) F=59 (55%)	PFJ OA Mean age 55.5 years K-L score 2/3 PFJ symptoms (stairs/ rising from chair) 3 months of daily pain VAS >40/100. BMI=30.7	Brace worn for 7.4 hours a day on average for 6 weeks.	No brace	Isometric MVC to assess quadriceps muscle strength and AMI.	At 6 weeks: between group difference in MVC (9.09 Nm; 95%CI: −4.89 to 23.07) between group difference in AMI −8.62%; 95% CI: -13.90% to -3.33%)
**Crossley et al. [**16**]**	Within subject design with randomised order.	n = 14 M=2 (14%) F=12 (86%)	Predominant PFJ OA. Mean age 56.9 (SD 7.4). Mean BMI 27.6 (SD 3.4). Anterior knee pain with stairs, squatting, rising from sitting. Tenderness peripatellar region. Radiographic evidence of osteophytes or severity grade ≥2	Tape	No tape	Patella position on MRI. VAS (pain on performing single leg squat x5).	Immediate pre post rape within groups mean differences patella lateral displacement (2.94% 95%CI: 0.37 to 5.51 p=0.028) Bisect offset: 0.58% (95%CI: -3.35 to 4.5 p=0.757) patellar lateral tilt angle: -3.57^0^ (95%CI: 2.14 to 4.19 p<0.001) VAS pain: -15.3mm, 95%CI: 0.4 to 30.3 p=0.045).
**Crossley et al. [**17**]**	Randomised Controlled Trial.	n = 92 M=39 (42%) F=53 (58%)	PFJ OA. Mean age: 54.5 (SD 10 years). BMI: 27.6 3/10 pain VAS during PFJ loading activities (using stairs/rising from sitting or squatting) and on most days in the past month. Evidence of PFJ osteophytes on radiograph.	PFJ targeted programme that combined exercise, education, manual therapy and taping. 8 treatments of 60-minute duration over a 12-week period.	Control group: physiotherapist delivered, single-patient osteoarthritis education. 8 treatments of 60-minute duration over a 12-week period + home exercises	Perceived GRoC, VAS pain during aggravating activity. KOOS	At 3 months: superior GRoC outcomes intervention group (much improved n = 20/44): control (much improved: n = 5/48). VAS (mean difference -15.2mm 95% CI: -27 to -3.4) KOOS –ADL (mean difference 5.8; 95% CI: −0.6–12.1) KOOS pain (mean difference 6; 95%CI: 0.1 to 12.6) KOOS symptoms (mean difference 3;96%CI -3.1 to 8.9) KOOS sport (mean difference 8.7; 95% CI -1.2 to 18.6) KOOS QoL (mean difference -0.1; 95% CI: -7.1 to 7) No significant VAS or KOOS differences at 9 months.
**Cushnaghan et al. [**18**]**	Within subjects crossover trial with randomised order	N = 14 F=10 M= 4	Anterior knee pain on walking and with using steps and stairs. Radiographic PFJ OA predominant in lateral facet. Concomitant TFJ OA in all subjects. Mean age 70.4yrs (range 55–84) Disease duration 12.5yrs	1. Medial directed patellar taping 2. lateral directed patellar taping 4 days for each tape condition	Neutral taping 4 days for each tape condition	VAS knee pain	At day 4: Mean difference neutral v medial tape =15.5mm (95%CI 2.4 to 28.6, p=0.023) Mean difference neutral v lateral tape at day 4 = -8mm (95%CI -22.5 to 6.5)
**Kumar & Ganesh [**20**]**	Randomised trial	n = 60 M/F not stated	Radiographic PFJ OA with anterior knee pain VAS knee pain equal and greater than 3cm Age not stated	Short wave diathermy + joint mobilisations + isometric exercises + medial patellar taping	Short wave diathermy + joint mobilisations + isometric exercises + lateral patellar taping	Knee pain VAS WOMAC	Lateral taping group “highly significant” compared to medial taping group for VAS and WOMAC (p=0.0001)
**Hunter et al. [**19**]**	Randomised crossover trial	n = 80 M=17 (21%) F=63 (79%)	Lateral PFJ OA or mixed lateral PFJ with concomitant TFJ OA but demonstrates source of symptoms is PFJ with anterior knee symptoms on most days with stair climbing and/ or rising from a chair and patellar mobilisations. Mean age: 60.5 (SD 9 years) BMI 27.6	Active treatment (Treatment B): BioSkin Q Brace with realigning T-strap for 6 week duration and mean of 4.8 hours/day, followed by a washout period (6 weeks) and then crossover to 6 weeks of brace with no strap (Treatment A).	Control (Treatment A): BioSkin Q Brace without realigning T-strap for 6 week duration and mean of 4.3 hours a day, followed by a washout period (6 weeks) followed by 6 weeks of wearing a brace with strap (Treatment B).	Primary: VAS (average pain over previous week). Secondary: WOMAC (pain, function, stiffness subscales).	At 6 weeks: No significant brace treatment effect (VAS −0.68, 95% CI: −6.2 to 4.8 *p*= 0.81) No significant difference between the groups for WOMAC pain, function or stiffness subscales.
**Quilty et al. [**21**]**	Randomised controlled trial	n = 87 M/F not stated	Chronic knee pain with predominant PFJ OA on radiographs (PFJ osteophytes). Mean age: 66.8 years (SD 9.5) BMI:30	Physiotherapy delivered treatments (exercises, patellar taping, footwear and postural advice). 9 x sessions, 30-minute duration over 10 weeks.	Control (no treatment)	Primary: VAS (overall pain during past month). WOMAC function sub-score. Secondary: MVC to assess quadriceps strength.	At 5 months: adjusted between means differences: VAS -6.4mm (95% CI: -15.3 to 2.4) WOMAC -0.6 (95% CI: -3.7 to 2.4) no significant differences between groups at 12 months quadriceps muscle strength at 5 months 11.7Nm (95% CI: 4.5 to 19; p = 0.002) but not 12 months (p=0.08).
**Tan et al. [**22**]**	Randomised controlled trial	N = 26 F 16	Clinical diagnosis of PFJ OA based on NICE guidelines. Mean age 60 (SD8)yrs	Commercially available foot orthotics 6 weeks continuous wear	Sham foot orthotic inserts 6 weeks continuous wear	Primary: feasibility of full RCT Secondary KOOS AKPS VAS GRoC	Adjusted mean difference (95% CI) 6 weeks: KOOS pain: 8.1 (-6.9 to 23.1) KOOS symptoms: 4.4 (-6.6 to 15.5) KOOS ADL: 13.7 (0.2 to 27.2) KOOS Sport: 25.7 (-1.7 to 53) KOOSQoL: 11.3 (-1.4 to 24) AKPS: 9.1 (− 8.6 to 26.8) VAS most aggravating activity: 21.9mm (2.1 to 46.0) Average VAS on most aggravating activity: 15.8mm (− 4.9 to 36.6) GRoC foot orthoses group: median value 2.5(min= -1; max = 6) GRoC sham group: median value 3(min=0; max= 6)
**Tan et al. [**23**]**	within-subject, cross-over design with randomised order	N = 21 F=14	Clinical diagnosis of PFJ OA based on NICE guidelines mean age 58 (SD8) yrs BMI 27.0 (SD 4.8)	Commercially available foot orthotic	Sham inserts	Primary outcome: biomechanical motion effects Secondary outcome VAS pain	No significant immediate effects of foot orthotics compared to sham inserts on VAS pain scores VAS mean differences (95% CI) Level walking 4.2 (-2.9, 11.2) Stair ascent −3.4 (-13.1 to 6.3) Stair descent 0.7 (-11.5 to 12.9)

Abbreviations: *AMI* arthrogenous muscle inhibition, *MRI* magnetic resonance imaging, *OA* osteoarthritis, *PFJ* patellofemoral joint, *BMI* body mass index, *MVC* maximum voluntary contraction, *GRoC* perceived global rating of change, *SD* standard deviation, *K-L* Kellgren-Lawrence, *VAS* visual analogue scale, *KOOS-(ADL)* knee injury and osteoarthritis outcome score (Activities of Daily Living), *WOMAC* Western Ontario and McMaster Universities Osteoarthritis Index, *M* males, *F* females, *KOOS QoL* knee injury and osteoarthritis outcome score (Quality of Life), *NICE* National Institute for Clinical Excellence

### Taping interventions

The effects of patellar taping were assessed by a trial of good quality [16] and fair quality [18]. Both had cross-sectional designs studying the immediate or very short-term effects only. Crossley et al. [16] compared 14 PFJOA patients with 14 healthy subjects. For the purpose of this review, we extracted the within-subjects’ data for tape versus no-tape in the PFJOA group only, as there was no comparison between those with PFJOA and the healthy control group. The intervention was two pieces of rigid sports tape that applied pressure to direct the patellar medially and superiorly. Another two pieces of tape were applied distal to the patella to unload the infrapatellar fat pad. The tape was applied for 1-day testing only. Compared to no tape, taping significantly changed patella lateral displacement (2.94% 95%CI: 0.37 to 5.51, p=0.028) and patellar lateral tilt angle (−3.57° (95%CI: 2.14 to 4.19, p<0.001). The difference between tape and no tape in mean VAS pain score immediately after performing a single-leg squat was −15.3mm (95%CI 0.4 to 30, p=0.045); this was a statistically and clinically meaningful decrease in pain within subjects with PFJOA.

Cushnaghan et al. [18] pulled a strip of rigid sports tape across the patellar medially and compared this to lateral directed tape and then to a piece of tape put across the patella with no directional pull (neutral). After 4 days of treatment, there was a clinically meaningful and statistically significant reduction of pain for medial directed taping compared to lateral or neutral taping (mean difference neutral v medial tape = 15.5 (95%CI 2.4 to 28.6), but not for neutral v lateral tape = −8 (95% CI −22.5 to +6.5).

### Bracing interventions

Four studies assessed the effect of bracing. A randomised crossover trial [19] compared the effects of two 6-week periods of brace wearing with and without a realigning patella strap in 67 participants with PFJOA. The daily average self-reported time for brace wearing for either brace was between 4.3 and 4.8 h. There was no significant difference between brace with and without the strap in reduction in VAS pain (0.7, 95% CI: −6.2 to 4.8; p= 0.81) nor in WOMAC pain (−0.11, 95%CI: −0.66 to 0.88; p = 0.77), stiffness (−0.11, 95% CI: −0.53 to 0.32; p= 0.61) or function (−0.02, 95% CI: −2.83 to 2.79; p= 0.99) for the two treatment periods.

**Fig. 2:**
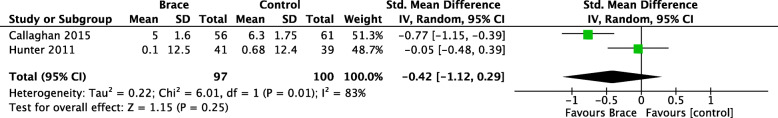
VAS for brace trials versus controls

**Fig. 3:**
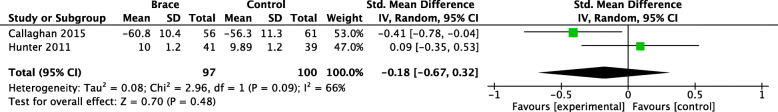
KOOS PAIN/WOMAC pain brace trials versus controls

Callaghan et al. [13] performed a 6-week RCT to examine the effects of bracing versus no bracing on bone marrow lesions (BML) and pain in 126 PFJOA patients. The daily average self-reported brace wearing time was 7.4 h. The brace group experienced a clinically and statistically significant decrease in VAS for a nominated aggravating activity (−13mm, 95%CI: −20 to −7; p<0.001) and reduction in BML volume in the PFJ compartment on magnetic resonance imaging (MRI) −490.6mm^3^ (95% CI: −929.5 to −51.7) (p=0.03). Although both trials used the same outcome after the same 6-week period of brace wearing, there was evidence of heterogeneity of the trials for the VAS (I^2^ = 83%, p = 0.02). The data were pooled using a random effects model of the standardised mean difference and showed no overall statistically and clinically significant benefit on a visual analogue scale (VAS) (standardised mean difference (SMD) = −0.42 (95%CI −1.12 to +0.29; Fig. 2). For the same two trials, the KOOS pain and Western Ontario and McMasters (WOMAC) were not significantly improved by bracing (SMD −0.18: 95%CI −0.66 to 0.31; Fig. 3).

Two further studies used data from the latter trial to look at the effect of bracing on quadriceps muscle strength and inhibition [15] and patellofemoral alignment [14]. There was a significant reduction in muscle inhibition in the brace group compared to no brace at 6 weeks (n = 106 between-group difference, −8.62%; 95%CI: −13.90% to −3.33%; p=0.002) with no significant change in quadriceps strength [15]. A further 30 patients from the same cohort were randomised to brace and no brace to assess the effect on structural patellofemoral parameters, using weight bearing MRIs to assess patellar position and patellofemoral alignment [14]. The application of a brace compared to no brace significantly increased PFJ lateral contact area (n = 30 within subjects difference 0.94cm^2^ 95% CI 0.07 to 1.81, p=0.04) and reduced PFJ lateral distance on weight bearing MRI (n = 30 within subjects difference 0.06cm 95% CI 0.12 to 0.01, p=0.03) which was assumed to reduce patellofemoral contact stresses.

### Foot orthotics interventions

Two good quality studies by the same research group examined the effects of foot orthotics on painful PFJOA. A RCT [22] compared a commercially available foot orthotic to a flat insert sham. The purpose of this trial was to provide feasibility information on sample size, recruitment for a full scale trial. The active intervention orthotic fitted inside the shoes had a 6° varus wedge with a medial arch support. After 6 weeks both groups reported improvements in pain and function. The foot orthotic group demonstrated a greater but non-significant improvement in mean change in maximum and average pain severity on a VAS during the most aggravating activity (of either ‘rising from sitting’, ‘stair ambulation’, or ‘squatting’) in the previous week (maximum VAS 21.9mm (95%CI: −2.1 to 46.0); average VAS 15.8mm (95% CI − 4.9 to 36.6)). Although not statistically significant, the between groups VAS scores exceeded the minimal clinically important difference for chronic musculoskeletal pain. The Knee injury and Osteoarthritis Outcome Score - activities of daily living (KOOS-ADL) subscale was significantly improved in the foot orthotic group compared to the sham group (13.7; 95% CI 0.2 to 27.2). There were no significant between groups adjusted mean differences in the other KOOS subscales (Table 2) including KOOS pain (8.1; 95% CI: −6.9 to 23.1). The second good quality trial [23] compared the immediate effects of foot orthotics and flat sham inserts on lower limb biomechanics, knee pain and confidence in individuals with PFJOA. For the immediate effect on VAS knee pain, there were no statistically or clinically significant differences between the foot orthotic and the sham insert during level walking (4.2mm, 95% CI −2.9 to 11.2), during stair ascent (−3.4mm, 95% CI −13.1 to 6.3) or during stair descent (0.7mm, 95% CI −11.5 to 12.9).

### Multimodal physiotherapy interventions

Three RCTs studied the effects of multimodal treatment programmes delivered by physiotherapists in patients with PFJOA. The study quality ranges from good [17, 21] to poor [20]. Quilty et al. [21] compared the effect on pain and function of a combination of exercises, patella taping, posture and footwear advice (9 sessions, 30-min duration over 10 weeks) with a control group. Those randomised to the control group were not informed that they were in a trial. At the baseline visit, patients in both the intervention and control groups had a half-hour discussion with a physiotherapist concerning diagnosis, prognosis, footwear, weight reduction and activity. General exercise was encouraged but no specific quadriceps exercises were advised. Kumar and Ganesh [20] gave all participants the electrical therapy modality of short wave diathermy, combined with joint mobilisations and isometric exercises. One group was randomly assigned to medial patellar taping and the second group to lateral patellar taping. The shortwave diathermy was applied for 20 min daily, but further details of the settings are not provided. The isometric exercises were to the quadriceps in supine, maintained for 6 s and repeated for 10 times with 10 s rest between each repetition. The taping technique was described as displacing the patella medially or laterally using manual pressure and then maintained in this position by tape across the middle of the patella using light to moderate pressure. Data for the taping intervention could not be extracted for separate analysis. Crossley et al. [17] compared combined exercises, patella taping, manual therapy and education with a control group of education only single-patient sessions, designed to control for the patient therapist interaction and psychosocial contact inherent with the PFJ-specific combined physiotherapy treatment (8 sessions, 60-min duration over 12 weeks).

Quilty et al. [21] found no significant between groups difference in disability on the WOMAC function subscale after 5 months. Kumar and Ganesh [20] described the analysis for WOMAC between group A and group B after 9 months as highly significant (p= 0.0001), but did not provide any further details such as means and variance. There was no reference to the clinical significance of the outcome. Crossley et al. [17] noted no significant between groups improvement in the KOOS-ADL.

Quilty et al. [21] found no statistically or clinically significant reduction in average ‘pain during the previous month’ (VAS −6.4mm) at 5 months. Crossley et al. [17] recorded a clinically meaningful and significant between groups mean reduction in ‘pain during an aggravating activity’ (VAS −15.2mm) at 3 months.

**Fig. 4:**
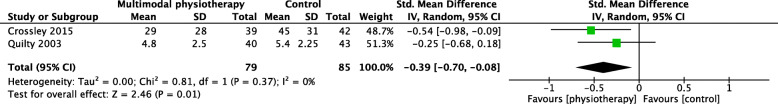
VAS multimodal physiotherapy versus controls

Pooling the data from the two good quality trials which used taping, with a random effects standardised mean difference, there appeared to be evidence of a small overall beneficial effect of the intervention for the VAS (−0.41 95%CI: −0.71 to −0.09; Fig. 4).

## Discussion

Overall this systematic review found good quality evidence that a combined physiotherapy approach may cause a reduction in patellofemoral pain. It is clear that more robust trials are needed to better define the role of these therapies as well as bracing, taping and foot orthotics in the management of symptomatic PFJOA. The consistent flaws in the good quality trials were the lack of blinding of the therapists in all the trials and the lack of blinding of the participants in all but two trials.

A greater number of participants in the reviewed trials were women. This is consistent with data concerning the frequency of PFJOA in the general population. There are no standardised clinical methods used to diagnose PFJOA [29]. Nine studies used a combination of subjective and objective information and radiographic evidence of PFJOA to make the diagnosis. Two based their assessments on NICE clinical guidelines which stipulated that imaging was not required for a clinical diagnosis of OA.

Nine studies used pain on stair climbing and rising from a chair as a subjective criterion and four studies also included pain on a squatting manoeuvre. Tan et al. [22, 23] used NICE clinical guidelines which included the criteria of anterior knee pain greater than 30/100cm on the VAS during stair ambulation, sitting or squatting. These activities are known to load the PFJ and to be painful activities in PFJOA. Quilty et al. [21] did not use any specific subjective indicators commonly used to diagnose PFJOA. Their participants were identified by chronic knee pain and radiographic evidence of PFJOA. Pain reproduced when palpating the patella facets was used as an objective assessment of PFJOA in 4 of the studies [13–16]. Although not validated as a diagnostic test there is general consensus within the literature that this technique is useful in characterising PFJOA [29, 30].

Using the same outcome measure allows comparison of a treatment modality’s effectiveness across the different trials. Although a pain VAS was used in 9 out of the 11 studies, each study recorded the pain score differently, namely performing a single leg squat [16], during an aggravating activity [17, 22], average pain in past week [19], average pain during the past month [21] and average pain during a patient nominated aggravating activity [13]. Additionally, studies had different time points for assessment.

Four good quality studies examined the effect of bracing in participants with PFJOA, though three of these were based on data from a single trial. All these four studies used the Bioskin Q brace which means the results are only applicable to this specific brace and cannot be generalised to all knee braces. Hunter et al. [19] found no significant treatment differences between wearing a brace with a strap compared to a group wearing a brace without a strap for an average of over 4 and less than 5 h a day. Participant preference dictated whether the patella strap was used. Conversely, Callaghan et al. [13] noted a clinically and statistically significant reduction in pain after wearing a brace compared to a no-brace control group for an average of 7.4 h daily for 6 weeks. The difference in daily time wearing the brace and the absence of a no-brace control group in Hunter et al. [19], and the different VAS questions used in the studies may potentially explain these two trials’ differing results.

Patellar taping is a well-established intervention for non-arthritic patellofemoral pain (PFP) or anterior knee pain [31]. It is inexpensive, and after instruction by a clinician, is self-applied by patients. Its mechanism is still unclear, but reviews have reported its pain reducing effects in PFP [32–34]. Cushnaghan et al. [18] were the first to study taping for PFJOA in their small (n=14) crossover trial of three different forms of patellar taping. Since then, there has been only one other study [16] of 14 participants on efficacy of taping for PFJOA. This good quality study found a significant immediate reduction in pain on the VAS (−15.3mm). As taping is known to loosen over time, it is unknown whether this effect would continue in the medium or longer term. The two good quality multimodal intervention studies delivered by physiotherapists [16, 21] included a patellar taping component but due to the combined approach the isolated effects of the taping modality cannot be extrapolated. Further, high-quality studies assessing longer-term effects of taping are required.

Owing to the multi-factorial nature of painful PFJOA, a combination of treatment modalities is often selected to address the different dimensions of pain and dysfunction. Overall, there was evidence of a reduction in pain among those randomised to a multimodal intervention but no significant improvement in self-reported physical function at 3 months [21] and at 5 months [17]. However, it is unclear whether participants had continued with their exercise programme once the treatment sessions had ceased and this may explain the lack of improvement in the long-term results. Additionally, Crossley et al. [17] had a high dropout rate of 21% at the 9-month follow-up.

The results of the review highlight the lack of standardisation in clinical trials of PFJOA. In order to advance our understanding of treatment response in this area, clinical markers and objective tests to diagnose PFJOA should be standardised. Additionally, there should be a consensus on the tool used to score PFJOA on radiographs. Due to the multi-factorial nature of PFJOA, further research is required to examine the effect of targeted physiotherapy interventions and where possible, methodologies and outcome measures should be standardised. The same validated outcome measures should be used across these clinical trials in order to allow comparisons of the treatment modalities. Long-term effects should also be examined.

### Limitations

There were some limitations to our review. The eleven randomised clinical trials were of poor, moderate and good quality for taping, bracing and foot orthotics. There were none of excellent quality. One major limitation in all the trials was the lack of blinding of the therapists delivering the treatment, and only three trials were able to blind the participants to treatment allocation. In the eight other studies, it is likely participants were able to distinguish between the interventions. The assessors were blinded to group allocation in seven out of the eleven studies which would mitigate against, though not exclude the possibility of bias. The included papers were in the English language only; therefore, studies published in other languages may have been missed. This review has not been registered online.

## Conclusion

There was a relative paucity of trials studying the effect of a biomechanical device therapy in patients with symptomatic PFJOA. There is some good quality evidence that a combined physiotherapy approach significantly reduces short-term pain in those with PFJOA. Long-term effects of all interventions are still unknown, which indicates the need for further research to determine the longer term impact of all biomechanical devices on outcomes in symptomatic PFJOA.

## Supplementary information

**Additional file 1:.** MEDLINE search strategy – October 2020

## Data Availability

Data are available from the corresponding author on reasonable request.

## Abbreviations

AMI: Arthrogenous muscle inhibition
MRI: Magnetic resonance imaging
PFJOA: Patellofemoral joint osteoarthritis
OA: Osteoarthritis
PFJ: Patellofemoral joint
BMI: Body mass index
MVC: Maximum voluntary contraction
GRoC: Perceived global rating of change
SD: Standard deviation
K-L: Kellgren-Lawrence
VAS: Visual analogue scale
KOOS-(ADL): Knee injury and osteoarthritis outcome score (Activities of Daily Living)
WOMAC: Western Ontario and McMaster Universities Osteoarthritis Index
M: Males
F: Females
KOOS QoL: Knee injury and osteoarthritis outcome score (Quality of Life)
NICE: National Institute for Clinical Excellence
