# Supplementary material for: Management of patellofemoral joint osteoarthritis using biomechanical device therapy: a systematic review with meta-analysis
Source: Syst Rev. 2021 Jun 9;10:173. doi: 10.1186/s13643-021-01708-3 (PMC8191025; doi:10.1186/s13643-021-01708-3)
Supplement: Supplementary file 1 — Additional file 1:. MEDLINE search strategy – October 2020 [file 13643_2021_1708_MOESM1_ESM.docx]

|  | MEDLINE search strategy – October 2020 |
| --- | --- |
| Population | 1. Osteoarthritis  2. OA  3. arthritis  4. arthrosis  5. arthroses  6. patellofemoral joint  7. PFJ  8. patello-femoral  9. PFJOA  10. 1 or 2 or 3 or 4 or 5 or 6 or 7 or 8 or 9 |
| Intervention | 11. brace  13.bracing  14. taping  15. tape  16. strapping  17. insole  18. insert  19. orthosis  20. orthoses  21. orthotic  22. 11 or 12 or 13 or 14 or 15 or 16 or 17 or 18 or 19 or 20 or 21 |
|  | 23. 10 AND 22 |
| Limits | limit 23 to English language and human studies |
